# Preliminary Study on PCC-Chitosan’s Ability to Enhance Microplastic Excretion in Human Stools from Healthy Volunteers

**DOI:** 10.3390/foods14132190

**Published:** 2025-06-23

**Authors:** Claudio Casella, Umberto Cornelli, Santiago Ballaz, Martino Recchia, Giuseppe Zanoni, Luis Ramos-Guerrero

**Affiliations:** 1 Department of Chemistry, University of Pavia, Viale Taramelli 12, 27100 Pavia, Lombardy, Italy; icarocus@gmail.com (C.C.); gz@unipv.it (G.Z.); 2 School of Medicine, Loyola University, Chicago, IL 60660, USA; ucornelli@gmail.com; 3 School of Medicine, Loyola University, 20129 Milan, Lombardy, Italy; 4 Faculty of Health Sciences, Universidad del Espiritu Santo, Samborondón P.O. Box 09-01-952, Guayas, Ecuador; sballazg@gmail.com; 5 Mario Negri Alumni, 20156 Milan, Lombardy, Italy; statmed@hotmail.com; 6 Grupo de Investigación en Bio-Quimioinformática, Carrera de Ingeniería Agroindustrial, Facultad de Ingeniería y Ciencias Aplicadas, Universidad de Las Américas (UDLA), Quito 170513, Ecuador

**Keywords:** microplastics, chitosan, *Procambarus clarkii*, human stools, human health, food supplement

## Abstract

Recent studies have indicated that microplastics (MPs) may accumulate in the human body, potentially posing health risks. This preliminary study aimed to investigate the effect of a food supplement (FS: 0.8 g of chitosan derived from *Procambarus clarkii*, PCC) on the fecal excretion of MPs (20–500 µm size) following ingestion of a standardized meal (SM). Ten healthy volunteers (non-smokers, non-drinkers, non-drug users) participated in a two-phase, crossover design conducted one week apart. In both phases, participants consumed an SM after overnight fasting, and fecal samples were collected the following morning (7–10 am). Phase 1 served as baseline (no PCC), while in Phase 2, PCC was administered immediately before the SM. Sixteen types of MPs were analyzed. A modest increase (5%) in fecal mass was observed after PCC intake. MP counts were 356 in the SM, 656 ± 110 in Phase 1 feces, and 965 ± 165 in Phase 2 feces. The excretion of nine MPs—PA, PAN, PAM, PE, PES, PET, PP, PS, and RA—was enhanced by PCC. These preliminary findings suggest that PCC promotes the fecal elimination of MPs. Further controlled studies are needed to validate these results and assess their potential relevance for dietary interventions.

## 1. Introduction

Increasing plastic production and consumption coupled with material cycle mismanagement has led to accumulating plastic debris. About 40 million Mt of plastic waste are expected to be produced by 2030 [1,2]. Microplastics (MPs) have entered the human food chain and are common air contaminants. In light of these aspects, one may define the present era as “the Plastocene”, and MPs can be considered as part of the particulate matter PM10 or PM2.5. MPs are consistent with polymers that are larger than 100 nm to 5 mm in size, whereas nanoplastics (NPs) are determined by polymers that are less than 100 nm. MPs/NPs are detected in several foods (e.g., seafood, honey, table salt, vegetables, fruit, milk, mineral water, etc.) [3]. The content in fruit, vegetables, or flour usually exceeds the quantity found in meat. Although the metabolic fate of these polymers is not fully understood, many of them have been found in the plasma at concentrations ranging from 0.5 µg/mL to 4.5 µg/mL [4]. MPs that are present in human urine [5] and kidney [6] have sizes ranging from 3 µm to 29 µm [7], which are outside of the NPs’ range. Stools also contain a considerable amount of MPs [8,9].

These contaminants are also found in olfactory bulbs [10], atheroma that result in cardiovascular events [11], during pregnancy [12], and are linked to a number of diseases. While controlling environmental MPs and NPs requires a very intricate and expensive procedure, lowering their absorption—at least through the gastrointestinal tract—seems to be easier. Stool examination is essential to ascertain whether ingested microplastics have been consumed entirely or nearly so and, subsequently, whether exposure to them has occurred. Due mostly to the difficulties in quantifying MPs and validating reliable methods, research on MPs in human stools is still in its infancy [13,14]. The process of breaking down and separating MPs from feces has led to the development of a number of techniques, such as different chemical and enzymatic treatments, to remove microplastics from feces. Nevertheless, MP quantification techniques are not yet harmonized or validated [14]. Limiting their absorption and increasing the excretion of MPs and NPs in stools may be achievable by using natural fibers that may bind these particles. A food supplement (FS), such as chitosan, a class of cationic polymers, serves as an illustration. They exhibit varying intrinsic viscosities due to the presence of primary amino groups (C2) and secondary hydroxyl groups (C6 and C3) that facilitate the binding of other polymers [15].

Louisiana crawfish (*Procambarus clarkii*) is an invasive species widely used as a biological control organism in many nations. *Procambarus clarkii*-derived chitosan (PCC) shows many advantages, including its biodegradability and biocompatibility, its safety for human consumption, and its ease to break down without leaving any harmful residues. Its capacity to bind to lipids and lessen fat absorption in the digestive tract (also known as weight control or cholesterol reduction) is another interesting benefit. It can also prolong meal freshness by delaying the oxidation of lipids [16,17,18,19]. In high quantities, PCC may cause allergies, change the flavor of food, or give it an unfavorable texture. Because PCC is only soluble in acidic conditions and incompatible with extremely alkaline meals, its use in particular foods could be limited [16,17,18,19]. Interestingly, in vitro studies have shown that PCC is able to bind MPs of different origin [20]. In this work, we investigated whether oral administration of PCC [21,22] to ten healthy volunteers would increase the MP excretion in stool after a standardized meal (SM) in the evening. The aim of the present in vivo experiment was to demonstrate the ability of PCC, used as a food supplement (FS), to bind and capture MPs within the human intestine.

## 2. Materials and Methods

### 2.1. Experiment Blueprint

**Figure 1:**
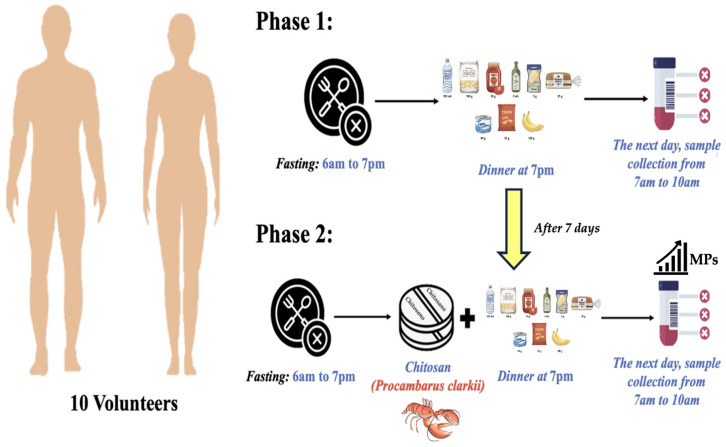
Scheme of the phases of experiment.

Inclusion criteria (healthy participants, free of shellfish or chitin derivative allergies) and exclusion criteria (individuals with gastrointestinal illnesses, chronic pathologies, or known chitosan allergies) were utilized for selecting the volunteers. Every volunteer received an informed consent form that included comprehensive details on the experiment’s aims, positive aspects, and dangers. Furthermore, the par-ticipant had all of their rights, including the capacity to leave at any moment without incurring any repercussions. All PCC administration and stool sampling procedures were carried out in compliance with GDPR guidelines to safeguard participant data. The Agency for Standard Supplements (IAPS) in Pescara, Italy, has approved the trials. Te code of ethics of experiments that the scientific committee has listed on the approval date of 16 September 2024; is the following: Code MNP-A1. According to the registry of the study, the experiment lasted for two weeks, with the first week referred to as Phase 1, and the second week as Phase 2 (Figure 1). During Phase 1, the diet was free for 6 days. On the day before the stool collection (day 7), the participants were instructed to fast and drink 1.5 L of water throughout the day until they had an SM to eat in the evening. The stool collection was requested the next morning between 7 and 10 am. Phase 2 was exactly the same as Phase 1, with the exemption of consuming 0.8 g of PCC right before the SM with half a glass of mineral water (an ingredient of the SM). The participants were requested to follow Phase 1’s free diet as strictly as possible. The researchers who attended the evening meal served the SM personally. A dosage of 0.8 g of PCC was chosen to reach a concentration of roughly 1 mg/mL after being mixed with stomach contents. It was believed that this amount would ensure enough viscosity and polymer charge to efficiently trap MPs without stressing the gastrointestinal tract. Considering that the average volume of human gastric contents after a normal meal is approximately 800 mL (it can vary between 600 and 1000 mL depending on the individual and the type of meal), 0.8 g was a reasonable amount of PCC. About 1 mg of PCC is required for every milliliter of stomach fluid in order to produce a gel that is sufficiently dense and rich in −NH_3_^+^ sites that are ready to interact with MPs. At lower doses (<0.5 mg/mL), PCC would remain excessively fluid with few −NH_3_^+^ groups accessible near microplastic surfaces. Excessive viscosity, a discernible slowing in the gastrointestinal transit, and possible discomfort during the swallowing or meal release process would be risks associated with higher doses (> 2 mg/mL). The 0.8 g of PCC is therefore well within the tolerance limits set for food consumption (up to 3 g/day without causing major negative effects) in terms of safety and bioavailability. At this concentration, PCC also creates a hydrogel that is stable in an acidic pH (−NH_3_^+^), which maximizes the efficiency of MP “entrapment” and maintains a relatively quick transit, with disposal within 12 h as required by the protocol.

**Figure 2:**
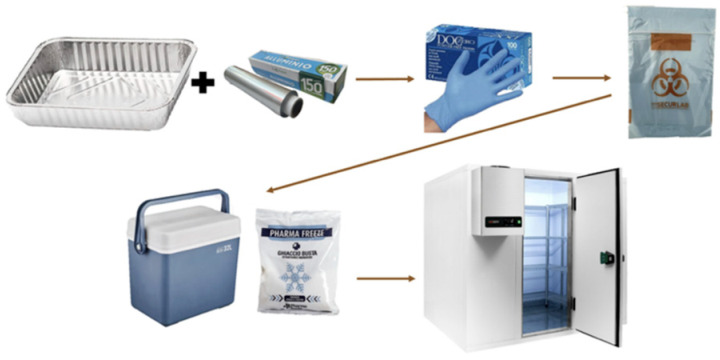
Scheme of stool sampling procedures.

The FS administered, also known as PCC, was food-grade chitosan derived from Procambarus clarkii (EU 2023/915) [22]. The intrinsic viscosity, determined by Huggins and Kraemer plots [23], was between 90 and 120 cPs (centipoize) and with a 90% deacetylation degree (DDA). According to the Mark–Houwink–Sakurada algorithm, the approximate molecular weight of PCC polymers was between 100 KDa and 140 KDa. Two stool collections were requested: the first for baseline values (Phase 1), and the second (Phase 2) one week after and following a single PCC administration at the dose of 0.8 g given in two capsules of 0.4 g each with half a glass of mineral water. The two experimental phases, shown in Figure 1, involved 10 volunteers. The stool collection was requested between 7 and 10 a.m. immediately following the evening SM. A basin (30 × 15 cm) was inserted into the toilet, abundantly covered with a 50 cm sheet of aluminum foil (Domopak, Volpiano, Italy) for the collection (Figure 2). At the end of defecation, the aluminum foil was recovered, closed with the hands, and placed in an airtight glass jar. Stools were weighed no later than 2 h, and the jar was maintained at −20 °C until analysis time. Regarding MP content, 5 g of stools were used, heated for 30 min at 35 °C, and then measured at room temperature. Since PCC is a natural fiber rather than the drug that was administered to otherwise healthy participants, a placebo group was not required.

### 2.2. Gathering Samples

The Department of Chemistry at the University of Pavia (Italy) conducted pretreatment analysis on stool samples, and the Faculty of Chemistry at the University of Oviedo (Spain) carried out SEM, FTIR, and stereomicroscope analysis, between October and November 2024. The experiment was carried out in the Faculty of Chemistry of the University of Oviedo (Spain), between October and November 2024. A total of 10 otherwise healthy volunteers, 3 females and 7 males, aged between 18 and 55 years, (non-smokers, non-drinkers, non-drug users) were included in the study (Table 1). Any pharmacological or FS treatment was in place. The volunteers were asked to fast during the day (only MPs-free water was permitted) and consume only the standardized meal (SM) during the evening before the morning stool collection. The investigators supervised the food intake during the whole daily sessions. The general characteristics of the volunteers are reported in Table 1.

### 2.3. MP Content and MP Characteristics in Each Food That Constitutes the SM

Each volunteer received 1.5 L of water free from MPs with the recommendation that they drink it all prior to the SM evening meal. The composition of water is reported in Table 2 and Table 3. The composition of the SM is displayed in Table 2. The products were purchased in the Italian market. Each participant consumed 730 g of food, which contained 359 MPs in the form of largely fibers and fragments (Table 2), since films were hardly present.

Table 2 displays a selection of items that are representative of a Mediterranean diet, which is popular in Southern European countries due to its high consumption and nutritional value. This diet includes a broad range of food matrices (solid, liquid, fat, protein, and plant-based), allowing the evaluation of the interactions between MPs in various physicochemical contexts. As one of the most well-studied and recommended diets in the world, any improvement in its food safety (such as lowering MPs) is relevant and of broad interest. Regarding the size, the data are compiled in Table 3, Figure S1 and shown as percentages of fibers and fragments.

### 2.4. SM Pre-Treatment

To prevent MP contamination, a glass microfiber filter (0.7 μm pore size, Whatman, Florham Park, NJ, USA) was used to filter all of the reagents and bifiltered distilled water. A beaker was used to hold each food item. To aid in stirring, 100 mL of bifiltered distilled water was added to the solid samples. In a flocculation tester (JLT6, VELPS Scientifica, Usmate Velata, MB, Italy), samples were agitated for 30 min at 120 rpm. It features a programmable timer (0–99 h), six glass beaker settings, an electrical speed regulating system (10–300 rpm), and a digital display. Each food item was then placed in 30 mL of a 50% H_2_O_2_ solution (VWR Chemicals, Briare, France) and allowed to sit at room temperature for 24 h. All organic compounds were then guaranteed to oxidize for 24 h at room temperature by adding 40 mL of Fenton reagent. Furthermore, a stainless-steel module with overlapping 500, 250, 100, and 20 μm sieves (CISA Sieving Technologies, Barcelona, Spain) was used to filter the material. The MPs that were captured on each sieve were cleaned and then collected in beakers using bifiltered distilled water. After that, a density separation method was used to separate the MPs from inorganic impurities using a ZnCl_2_ solution (d = 1.5 g/mL, 97% purity, VWR Chemicals, Briare, France). The MP solution was filtered using vacuum glass microfiber filters (0.7 μm pore, Whatman, Florham Park, NJ, USA). Every product that helps increase the SM had the same heat treatment.

### 2.5. Stools Pre-Treatment

**Figure 3:**
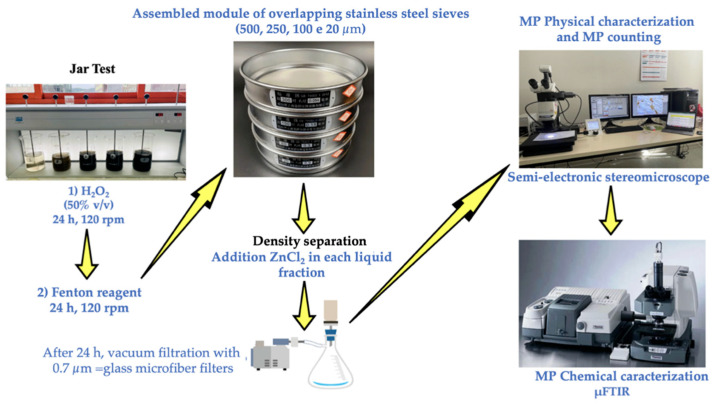
Scheme of the food and stool samples pre-treatment.

The identical protocol outlined in Section 2.3 of the present study, was also used to pretreat the stool samples (5 g wet weight for each sample) of the ten participants from both phases (Phase 1 and Phase 2, see Figure 3).

The arrows indicate the sequence of the process phases.

### 2.6. Microplastic Analysis

The MPs present in filters were counted using a semi-automatic stereomicroscope (Leica M205FA, Leica Microsystems CMS GmbH, Wetzlar, Germany) and a high-resolution color digital camera (Leica DFC310FX; 1.4 Mpixel, CCD, Leica Microsystems CMS GmbH, Wetzlar, Germany). The MP-fragment and MP-fiber diameters were also estimated using the Confocal UniOvi ImageJ program (Version 1.54p). The scanning electron microscope (SEM) analysis was used to assess the morphology of the MPs found in the food and stool samples (in each Phase) (JEOL-6610LV with microanalysis, Tokyo, Japan). SEM featured tungsten filament electron guns, a maximum resolution of 3.0 nm, and an operating voltage range of 0.5 to 30 kV. The range of magnification ranged from 5× to 50,000×. It used high-vacuum modes for samples with the best resolution and low-vacuum modes for wet or non-conductive surfaces. It made use of backscattered secondary (composition, topography, and shadowing) electron detectors. It had a 5-axis asynchronous mechanical eucentric stage that could handle samples up to 20 cm in diameter in addition to eucentric tilt and rotation. The SEM was completely automated, operated on a single PC, and automatically saved images in the BMP, TIFF, or JPG formats.

A µ-FTIR spectrophotometer (Perkin Elmer Spotlight 200i FTIR spectrophotometer, Springfield, IL, USA) from the Autonomous University of Madrid (UAM) Molecular Spectroscopy Unit was used to determine the MP’s chemical composition. For transmission analysis, MPs were positioned on supports (KBr pellets) that were transparent to infrared light. The results of an automatic examination of the generated infrared spectra were compared with a spectral database, which is stored in the previously mentioned apparatus and contains around 36,000 spectra of different compounds. The spectral range (550–4000 cm^−1^), resolution (16 cm^−1^), number of scans (30), and infrared beam aperture (20 × 100 microns for MP fibers and 50 × 50 microns for MP-fragments) were the measurement parameters used to study the MPs. A spectral database that included the spectrophotometer of more than 36,000 chemicals—including organic and inorganic substances, polymers, fibers, paints and derivatives, solvents, drugs, etc.—was used to compare the outcomes. The micrometric samples were examined using a Euromex Edu Blue(v2.4.9.0) magnifying glass (Duiven, Netherlands) with 20× and 40× magnification after the researcher obtained the sample count for each plate and the approximate size, shape, and color of each plate (data collected prior to analysis). Upon visual identification, the samples were manually placed on the surface of a produced KBr pellet (transparent in the mid-infrared region) and then placed in a designated sample holder of the infrared microscope. A magnifying lens was always utilized for this important task as a visual help. The micrometric samples were then handled using a tungsten needle tip or absorbent paper tips, which are most suited for microfibers with an electrostatic charge and are mainly employed in dentistry. For this stage of the pre-measurement process, the technician’s expertise was crucial. The sample container was moved to the infrared microscopy stage, where each micrometric sample was examined under a microscope after each plate sample had been deposited on the KBr pellet’s surface. The micrometric samples were analyzed infrared after the analysis process was configured with the previously mentioned measurement settings. By comparing the gathered spectra with the reference spectra kept in the measuring device’s Spectrum database, the MPs are identified. Some MPs may be released by plastic bottles and Falcon tubes used for sampling and sample treatment. The mean MP concentration released by plastic bottles was 1.33 ± 0.48 MPs/L after a triplicate analysis. These quantities are considered negligible, accounting for less than 1% of the total MP concentration detectable in each experiment [24,25,26].

### 2.7. Quality Assurance and Quality Control (QA/QC)

Quality control and assurance (QA/QC) was carried out from the time the MP samples were collected until they were quantified, in accordance with earlier studies [26]. Important QA/QC procedures included using glass microfiber filters (0.7 µm pore size), removing polymeric materials in the lab, and filtering chemical reagents before usage. Good field and laboratory practices (GLP) were used throughout the sampling and analysis process to minimize secondary contamination from MPs found in the air, on surfaces, and eventually on the equipment. Consequently, the sampling and processing samples were made using as little plastic as possible. When this was not feasible, procedural blanks were utilized. It was believed that a specific quantity of MPs might be released when Falcon tubes and plastic bottles were used for sample treatment and sampling. Control experiments were conducted in both situations to prevent any interference with the studies. The mean concentrations of MP emitted by Falcon tubes and plastic bottles were 0.5 ± 0.2 MPs/L and 0.4 ± 0.2 MPs/L, respectively. Because they accounted for less than 1% of the total MP concentration used in each experiment, their contamination was considered insignificant [26]. Each analysis and experiment was conducted in triplicate.

All food and stool sample filtration operations were carried out under close supervision in a certified laminar flow cabinet in order to avoid sample contamination. This kind of cabinet offers a clean-air working environment that meets ISO 5 standards, since it has a unidirectional flow of sterile, particle-free air. This device is commonly used as a part of MP contamination management methods. The following extra safety measures were observed in addition to operating in a laminar flow:

• Using fiber-free lab coats, face masks, and pre-washed nitrile gloves to prevent particles from being produced by the operator or clothing. It was recommended that all lab equipment, including forceps, funnels, and filters, be pre-washed with Type I ultrapure water before each use. Any leftover residue should then be dried using laminar flow.
• Blank controls: Procedural blanks, or control filters without samples, were processed alongside the experimental samples in order to evaluate any possible inadvertent contamination during the procedure.
• Keeping the filters in closed or protected systems at all times to minimize their exposure to the environment. These procedures offer a high degree of confidence that the MPs detected in the samples are exclusively from the ELs being examined and are not the consequence of contamination from external sources or artifacts of the experimental procedure.

### 2.8. Statistics

Due to the lack of prior data regarding MP fiber binding capacity, the number of subjects was determined heuristically. The U Mann–Whitney test was computed to ascertain the difference between MP count at baseline and following the administration of PCC. Data were presented as mean ± SD. Statistical analysis was performed using the SPSS software (version 24.0) for Windows (IBM, Armonk, NY, USA). Only 10 subjects were used to apply Cohen’s approach to the data, ensuring that any variable with statistical significance had a statistical power of at least 80%.

### 2.9. Ethics

The participants were required to sign a written consent form with a description of the product activity. Non-invasive testing was necessary and the medication was simply administered once per evening. Payment of any type was requested or given. The International Agency for Pharma Standard Supplements (IAPS) approved the experiment despite PCC actually falling under the food-grade product category [22].

## 3. Results

### Comparative MP Analysis Between Phase 1 (Baseline) and Phase 2 (PCC)

Table 4 lists the weight of the stools both before and after PCC therapy, together with the amounts of MPs recovered.

**Figure 4:**
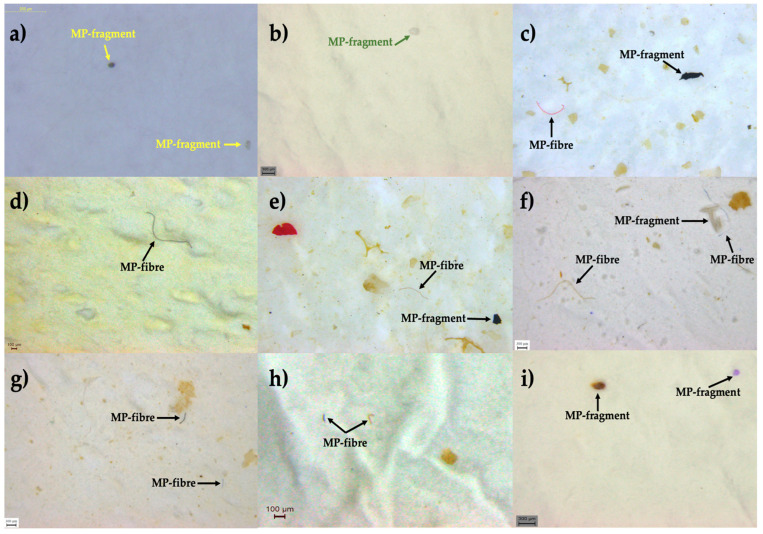
Stereomicroscope images of MPs identified in this study: from (**a**–**c**) (MPs in Phase 1 samples); from (**d**–**i**) (MPs in Phase 2 samples).

Using PCC resulted in a 45 ± 14% rise in MPs (U Mann–Whitney *p* < 0.05) and a 5 ± 4.2% increase in stool (U Mann–Whitney *p* < 0.05). Examples of MPs found and analyzed in stool samples are displayed in Figure 4.

Stool samples from the current study revealed 16 different types of MPs both before (Phase 1) and after 0.8 g of PCC was administered (Phase 2). The following MPs were analyzed: polyethylene terephthalate (PET), phenol formaldehyde (PF)_ resins, polypropylene (PP), polystyrene (PS), Teflon (PTFE), rayon (RA), acrylic fiber (AF), synthetic cellulose (CE), epoxy resin (ER), polyamide (PA), polyacrylamide (PAM), polyacrylonitrile (PAN), polyethylene (PE, particularly LDPE), polyetherimide (PEI), polyester (PES), and polyether urethane (PEUR). With considerable variations depending on the chemical composition of the MPs, the statistical analysis generally indicated a large rise in MP excretion following PCC, both unconditionally and substantially. Future research should increase the sample size, extend the sampling window, and use multiple comparison adjustments in order to validate these findings [13,14,20].

Table 5 shows the MPs % without distinguishing between fibers, fragments, and films.

The amount of MPs, which are shown in Table 6, was deemed more reliable for the statistical analysis than percentages (Figures S2–S4).

**Figure 5:**
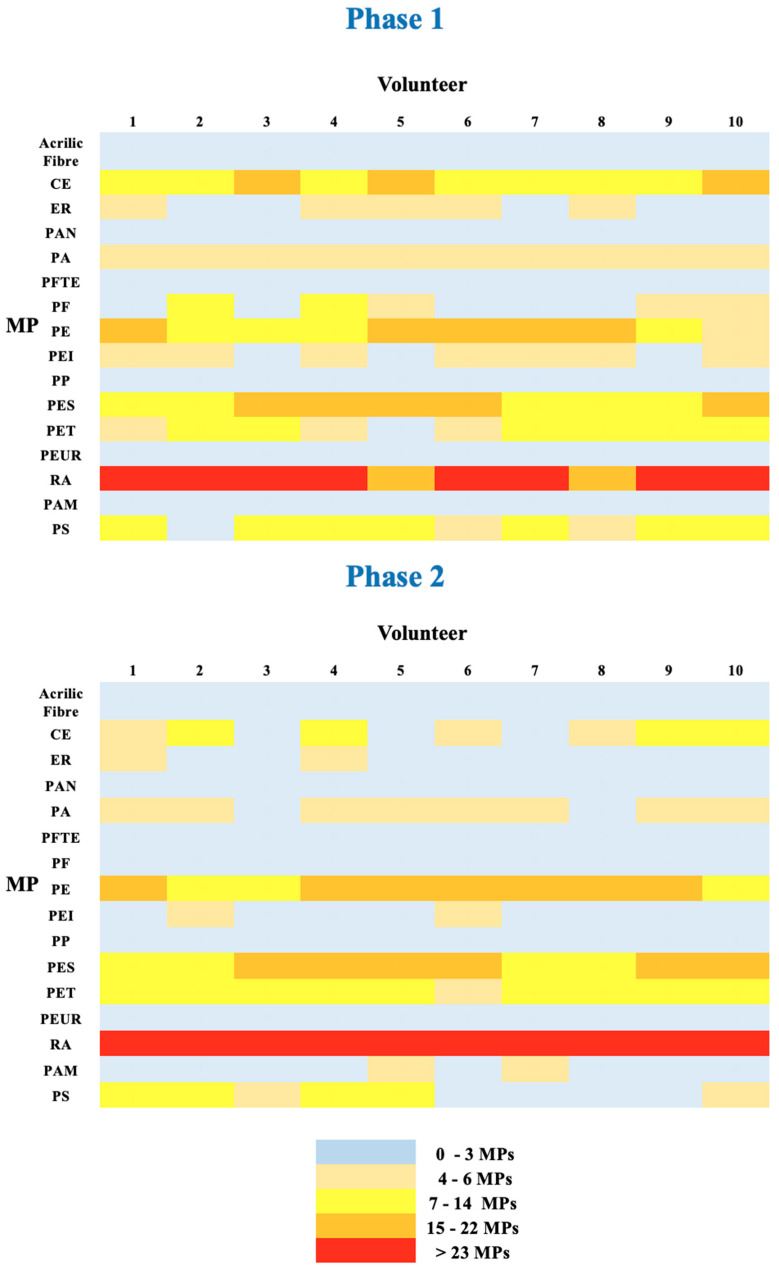
Heatmap Phase 1 and Phase 2.

**Figure 6:**
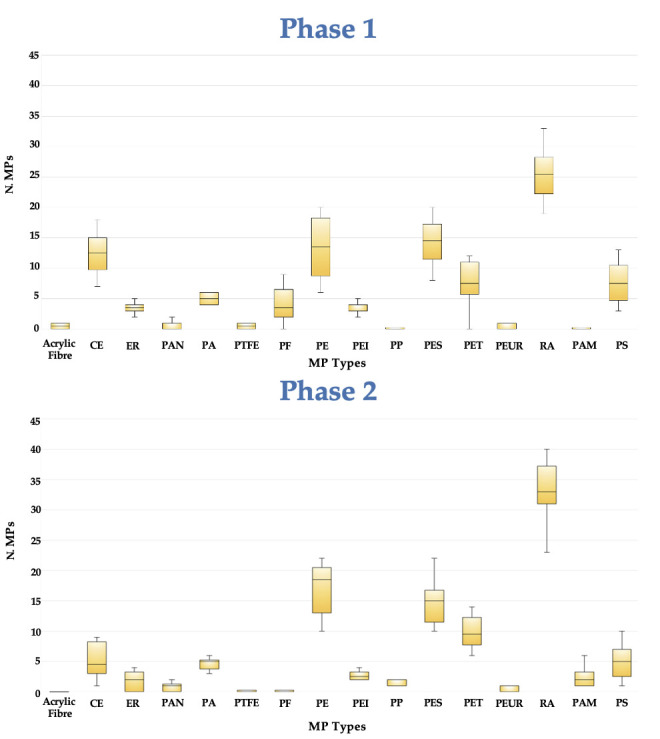
Boxplot Phase 1 and Phase 2.

All the healthy volunteers reported no adverse effects after receiving PCC. A heat map is included to help visualize the data gathered in both phases and aid in identifying any possible relationships between each of the MPs (Figure 5). Boxplot representations were created in order to identify outliers and examine the distributions of the different MPs in the ten volunteers in both periods (Figure 6).

Despite interindividual heterogeneity, PPC considerably enhanced the excretion of MPs in stool, especially PAM, PE, PET, PP, and RA (Figure 5).

In general, Phase 2 exhibits exhibited greater dispersion for several MP types (Figure 6), suggesting a higher degree of heterogeneity in the overall number of MPs found. When comparing some MP types, such as PE, PAM, RA, PP, and PET, where the number of MPs has risen. The median and dispersion revealed a statistically significant increase compared to Phase 1. PE and PAM in particular exhibited more variability and abundance with substantially higher medians and a broader interquartile range. Overall, Phase 2 tends to find more MPs in certain groups than Phase 1 did. This makes it clear that PCC administration was responsible for these modifications.

**Figure 7:**
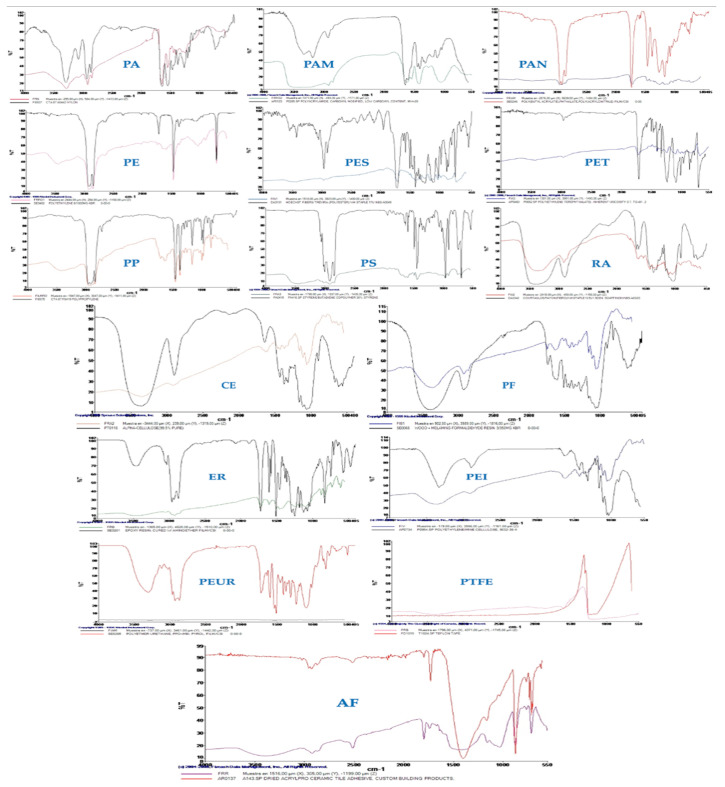
µFTIR images of MPs identified in the present study.

According to the data obtained between Phases 1 and 2, nine MPs (PA, PAN, PAM, PE, PES, PET, PP, PS, and RA; *p* < 0.05) showed a statistically significant increase in Phase 2. This suggests that PCC successfully captured these MPs (both fibers and fragments) in the gastric gel and moved them along the excretion path. The five other MPs that did not exhibit significant alterations (NS) were AF, ER, PTFE, PEI, and PEUR. These MPs may or may not show hydrophobic or electrostatic attraction for the PCC, depending on the circumstances. The delayed expulsion of the remaining two MPs, CE and PF, may be caused by a longer transit time or a stronger interaction with the PCC, as evidenced by the fact that they were excreted more in Phase 1 than in Phase 2 (*p* < 0.05). The presence of two MPs (PAM and PS) in the stools that were not present in the SM could have been caused by previous meals. Following ingestion, they most likely require at least 36 h (24 h ± 12 h) or longer to be excreted. The potential sources of the 16 MPs involved food-grade packaging, wrapping, and film (i.e., PE, PP, PET, and PS), disposable containers and utensils (i.e., PTFE, PS), fresh product packaging, paper and cardboard packaging (such CE), industrial paints, protective coatings (such ER), apparel, and technical materials (i.e., AF, PAN, PA, PES, and RA). Furthermore, the origins of the remaining MPs analyzed might have included materials used for coatings and adhesives (i.e., PF and PEUR), filtration/paper materials (PEI), and the usage of binders in specified food products (PAM) [16]. Figure 7 reports the 16 MPs found in the stool samples.

The differences among fibers, fragments, or films in each of the volunteers at baseline (Phase 1) and after PCC (Phase 2) are summarized in Table 7.

Fiber, fragment, and film excretion were significantly higher in Phase 2 compared to Phase 1 (U Mann–Whitney < 0.05). Regarding SM, MPs were divided into fibers (69%) and fragments (30%), films being almost absent (< 1%). The MP sizes in SM and stools are summarized in Table 8.

The percentages represent the pattern of excretion, which were almost identical in the two phases. For all the particle sizes, the values in Phase 2 were significantly greater than those in Phase 1 in terms of quantity. Following pH-dependent gelation of PCC, fibers (i.e., PA, PAN, RA, PES) are most commonly retained and carried to the colon, increasing their relative proportion in feces. Despite growing in absolute numbers, fragments represent a smaller portion of the total. PCC raises the percentage of smaller MPs (less than 200 µm) to about 85% by favoring their trapping and excretion. Alternatively, larger MPs (≥ 200 µm) are retained in smaller amounts, either due to their less effective capture or because they are kept in the gel network for a longer period of time. Interaction with fibers and small particles is made easier by the linear structure and high density of −NH_3_^+^ groups in protonated PCC, which causes them to become more “injected” into the gel matrix. Large films and fragments lose representativeness upon PCC, indicating that their geometry restricts entrapment or that they require more transit time to emerge from the gel. The study supports the findings that PCC administration not only raises the overall number of MPs expelled, but also alters their size and shape profile in feces in a number of ways.

The aforementioned statement describes how we were able to verify the results of the comparison of fecal samples taken before and after PCC administration, supporting the idea that PCC not only acts as a general “sponge” for MPs, but also has a tendency to more selectively trap specific types (fibers and smaller particles), changing the profile of what is eventually removed in stools (Figure S5).

## 4. Discussion

In this study, the quantity of MPs was assessed according to the most efficient analytical techniques [27,28,29,30,31] in order to determine the capability of PCC to increase MP excretion in stools 12 h after an evening meal (SM). The product was administered in a single dose of 0.8 g right before SM. It has been previously shown in vitro [32] that PCC may entrap various MPs in the 20–500 µm range. Furthermore, all of the plastic particles were linked to several chitosan polymers.

Nevertheless, there also were some limitations. The first concern was the atmospheric MPs in terms of PM10. The daily quantity of MPs was 1.68 mg to be added to 2.8 mg given with SM, considering that the WHO limit of 45 µg/m3 has not been exceeded and that the average adult subjects inhaled 15.7 m3/day of air. Even though the primary function of the human airways is “filtering,” and the gastrointestinal system is designed for “absorption,” nothing is known about MPs’ bioavailability in either situation. The settings of the two phases were considered equal in the present study in terms of total MP exposure, and the variation in excretion with stools can reliably be attributed to PCC activity. The second concern is SM, since certain foods with different brands may have higher or lower MP levels based on the ingredients and production lots. The third limitation is the time needed for some MPs to be excreted, which can take longer than 12 h. The mucus surrounding the enterocytes may hold certain MPs making their release considerably slower. The fourth issue is the significant variance of many MPs found in the stools of the volunteers. In this respect, this study should be regarded as an initial investigation that requires a greater number of cases and a longer period of treatment with PCC to confirm the data. Even when taking into consideration the most common MPs, the binding ability of PCC cannot be applied to all the plastic pollutants potentially present in foods and in the environment. Moreover, the chitosan family includes items with varying viscosities (molecular weight, MW). Some in vitro investigations have demonstrated that the results cannot be extrapolated to all brands because those with very low viscosities might not be effective. The last issue concerns the NPs that could not be found in foods or stools using the current analytical techniques.

Notwithstanding these limitations, the study produced several remarkable findings. The first observation was that MPs that were not present in the SM, were discovered in the feces This was the case of PS and especially PAM. The amount of PAM in feces after consuming PCC was substantially more than the amount excreted at baseline. PAM and PS were not present in the air of the town where the study was carried out [33]. This means that these particles were deriving from SM only. The second observation is that MPs provided with SM (359 particles), were evacuated with stool in Phase 1 and Phase 2 in significantly different amounts (656 and 965 particles respectively). The increased excretion compared to SM was the result of leftover MPs that were consumed at earlier meals. When 0.8 g of PCC was taken with the meal, the excretion of MPs increased by about 45%, but the volume of stools only increased by roughly 5%. If there is no distinction among MPs, it may be assumed that the elimination of 311 additional particles (965–654) accounts for 87% of those consumed during the SM. MPs of all sizes were significantly more excreted after PCC. With the exemption of a more consistent elimination of fibers >500 µm, the size distribution (fibers, fragments, and films) was similar to the baseline (Phase 1). PCC can serve as a “broom” in these situations, ensnaring “compatible” particles of each size and type (fibers, fragments, and films), and pulling them from the stomach up to the distal section of the gut and onward to the duodenum and ileum. The “compatible” MPs require a more detailed explanation.

From their fate in the volunteers’ stools to their consumption of SM and the administration of PCC, the dynamics of MPs are intriguing to speculate about. The volunteers ingested an average of 359 MP particles in the SM, mostly fibers and fragments with a size range of 20–500 µm. In order to ensure that the SM was the only significant source of MPs, MP-free water was supplied and the environment was controlled to reduce the quantity of MPs that could be inhaled. The PCC–MP blend (hydrogel) may be drawn by the colon, preventing particle absorption. The microbiota in the colon may break down the PCC to some extent, but the MPs remain unchanged and were eliminated in the stool together with unbound MPs from earlier meals. It is well known that the PCC polymers become soluble and flexible when the stomach’s pH reaches 2–3. This allows them to bind some of the chyme’s constituents, mainly lipids and starch, which are present in high concentrations, to form a number of very small networks. All of these tiny complexes have a propensity to gel as the pH rises in the ileum and duodenum. The MPs are particles that can get either entirely or partially trapped in this gel, reducing their bioavailability and allowing colonic bacteria to exploit them (at least partially) for growth and metabolism.

**Figure 8:**
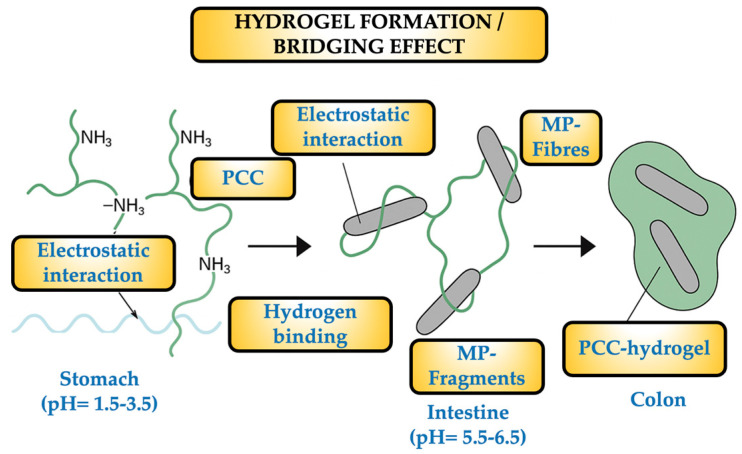
Hydrogel generation scheme.

When PCC (a copolymer of β-(1→4)-N-Acetyl-D-glucosamine and D-glucosamine, with DDA > 80%) dissolves in the stomach, its NH_2_ groups become significantly protonated. This causes the polymer to shift from being insoluble to soluble or colloidal, creating viscous solutions or scattered hydrogels that retain water and other tiny molecules. The surfaces of PES, PET, and PP fibers are comparatively hydrophobic and lack many polar groups. The fragments of PE and PS are identical and usually covered with impurities that provide polar sites, such as hydroperoxides and carbonyls. Therefore, even if they are still non-ionized at stomach pH, numerous carbonyl (C = O) and ester (–CO–O–) groups are able to form hydrogen bonds and provide hydrophobic interactions that are stabilized by the suspension’s aqueous phase. Simultaneously, MPs can adsorb ions (such as Cl- and Na+) and charged pollutants on their surfaces through indirect electrostatic interactions. In order to drag charged MPs or attached pollutants, protonated PCC can interact by drawing diffuse layers of ions. Stabilizing their connection, the PCC’s protonated and hydroxyl groups create bridges with the surface carbonyl groups and esters of the MPs. Polar and hydrophobic zones are present in the linear PCC chain in its partially deacetylated form; the latter can use Van der Waals forces to “wrap” or “knot” synthetic polymer fractals. Gel formation occurs in the duodenum (pH ≈ 5.0–6.0) and ileum (pH ≈ 7.0–7.5). As PCC travels through the intestinal tract, its solubility decreases, causing it to precipitate as a hydrogel. A three-dimensional matrix, or gel-fiber network, is formed during this simultaneous gelation, immobilizing the MP particles that were previously electrostatically attached. Physical entrapment (also known as a “molecular sieve”) can incorporate any free MP in the chyme into the gel. The colonic microbiota expresses chitosanase enzymes with limited capacity, which depolymerize PCC into oligomers but do not liberate MPs, which remain bound in the network. The MPs remain together until the very end, but the hydrogel loses cohesiveness during transit when the PCC’s glycosidic bonds break down. PCC oligomers and monomers (biodegraded or soluble) follow the usual route of fermented polysaccharides, and the MPs and PCC are evacuated together. When encapsulated or attached to gel fragments, the MPs (20–500 µm) cannot penetrate the mucosa and are expelled in the stool. Figure 8 illustrates the dynamic of the hydrogel generation and the “bridging effect”.

The arrows indicate the sequence of the process.

The bridging effect relies on the ability of each PCC chain to link to the surface of one fiber and “extend” to reach and adsorb another fiber if it is sufficiently long (usually having a molecular weight of 100–300 kDa). It is possible to stabilize a single PCC molecule on two, three, or more fibers simultaneously. The result is a network of interconnected fibers, joined into a single aggregate by a polymeric “bridge” at each junction. As more PCC chains adsorb, the size of the aggregates increases; several fibers knot together with multiple PCC linkages, increasing their diameter and decreasing their hydrodynamic mobility in the suspension. The hydrogel secures the aggregates in the intestine by encasing the entire assembly. The PCC/MP-fiber/MP-fragment aggregates stay bonded in the colon and are expelled as a visible “floc”. Accordingly, the bridging effect promotes the development of denser, more effective fibrillar aggregates in addition to increasing the total number of MPs ejected. While MPs/NPs have a virtually unknown future and are undoubtedly useless for caloric bacterial metabolism, starch and fat can be utilized as “fuel” by colonic bacteria. One may argue that one of the unknown factors contributing to dysbiosis is the microbiota’s capacity to construct and absorb MPs [34,35,36,37].

When comparing the findings of this study with those in earlier studies, the profile of MPs found in the stools can be regarded as comparable [8,13,14,17,26,38]. The most common MPs in those studies were PE, PP, PS, PVC, PET, and PA. Only one study found MPs like PU and PMMA [39]. Per gram of stools, these studies found amounts ranging from 3.5 MP/g to tens of µg of MPs [8,26,38]. The size range of the MPs evaluated in those studies was slightly wider (50–500 µm) than the current study (20–300 µm) [8,26,38].

**Figure 9:**
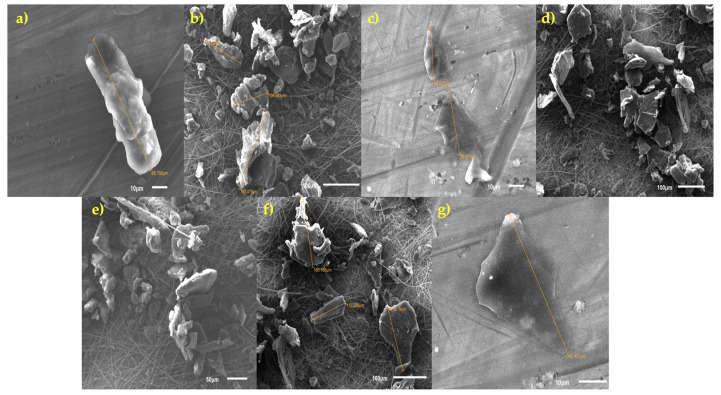
Illustrative SEM images of MPs identified in the stools, in the present study (Phase 2). (**a**–**g**) represent the different morphology shown by chitosan-MP complexes.

Among the 16 MP types that were examined included polymers with varying sizes, compositions of monomers, and shapes (fibers, fragments, or films). Despite the fact that electronic microscopy was documenting the binding for some of the MPs (i.e., PE, PET), all these factors make the binding of PCC very complex to explain. This was evident for very small and large monomer sizes (from 20 µm to 500 µm) at quantities compatible with those taken with a meal (1 g of PCC and 3 mg of each MP). In the current investigation, the particles belonging to PA, PAN, PAM, PE, PES, PET, PP, PS, and RA were more efficiently excreted following PCC because their size and monomer type were suitable for PCC binding. The effectiveness of hydrogel formation between PCCs and MPs could be evaluated using SEM data. Figure 9 reports the SEM images of the MPs found in the stool samples. Following their passage through the human digestive tract and subsequent expulsion, these SEM images of PCC-coated MPs make it clear that many of the particles have noticeably more uneven surfaces, with worn areas, grooves, and cavities that were absent from the original material prior to ingestion. This suggests that the MPs were affected mechanically (by peristalsis, friction with the fecal matrix) and/or chemically (by enzymes, an acidic stomach pH). Due to the observed chipped edges and smaller fragments (e.g., ~20–30 µm pieces) that separated from the 100–200 µm parent particles, there may be secondary fragmentation. The result could have different toxicological implications during intestinal transit. In some MPs, a more uniform layer was still present in other places, while in other MPs, the PCC appears to have detached or deteriorated (as shown by the loss of continuous texture).

This suggests that the coating could not be entirely resistant to bile salts, proteolytic enzymes, and fluctuating pH, among other digesting conditions. Additionally, regions where the PCC is best conserved were identified as points of organic matter accumulation, where groups of organic material (dietary fiber, mucus, and microbiota remnants) are preferentially attached. This is most likely because of the electrostatic affinity of the PCC or the development of “biolayers” (namely, biocoronas). Co-agglomerates of MPs were found in some locations, joined either together or with particles of organic matter to form aggregates that ranged in size from 100 to 200 µm. The exposure of gut flora, transit speed, and bioavailability may all be influenced by these associations. MPs are likely to stay “entangled” in the fecal matrix, which affects their retention and evacuation, according to the vast fibrous network (presumably mucopolysaccharides or undigested fibers). Overall, these SEM images provided an excellent understanding of how MPs physically changed and how long (or short) their PCC covering lasted after entering the human body. The possible release of NPs could be considered a risk factor for toxicological consequences. The observed fragmentation suggests that a nanofraction (<1 µm) with a highly unique absorption and toxicity profile may arise after passing through the gastrointestinal tract. This nanofraction is not apparent in scanning SEM at this scale. It would be prudent to use EDS analysis in addition to SEM to discover elemental indicators of PCC and validate its presence and composition after transportation.

A different condition can be established for CE and PF polymers, which were among the least abundant MPs in food and whose amounts in stools decreased following PCC administration {36]. CE is a linear polymer of glucose rings linked by acetal groups with intramolecular and intermolecular hydrogen-bonded hydroxyl, which renders chain rigidity. This structure can readily bind by PCC polymers, acting as a kind of anchor to limit MP absorption and delay excretion. The use of CE is common for many industrial uses (i.e., paper) and is abundantly present in vegetable and food packaging. Despite being composed into different structures (i.e., Bakelite, Atlanta, GA, USA; Novolac, Pocasset, MA, USA; Resols. Atlanta, GA, USA), PFs are all distinguished by their abundance of hydroxyl groups, which have the potential to bind PCC similarly to CE. The primary applications of this kind of polymer are as adhesives and binders for wood-based panels. In other terms, it will take more than 12 h for stools to expel the two final types of MPs. According to a matrix of gradual release, it is possible that PCC serves as a covering material for these final polymers. The only way to know if this will happen in these circumstances is to look at plasma levels.

The list of diseases most likely brought on by MPs or NPs grows nearly every week; thus, it is outside the purview of this investigation. Essentially, because of the extremely high production and use of plastics, our era could be referred to as “the Plastocene,” in which any disease that has not yet been identified can be linked to plastic contamination. It is not unexpected that MPs and NPs may prove protective for certain illnesses.

### Putative Limitations of This Study

Each one of the food items selected to set up the SM under investigation came from a single batch. This study underscored the significance of preliminary investigations and the necessity of further studies focusing on a wider variety of foods and volunteers. Complementary methods like high-resolution Raman spectroscopy (also known as atomic force microscopy–Raman or AFM–Raman) might identify particles smaller than 10 µm, enabling even more accurate characterization in later studies. One of the main drawbacks of the study was that stool collection was only carried out 12 h after SM. Some polymers may therefore need a longer transit time (>24–48 h) to be fully evacuated. Future studies should be carried out to assess the effect at lower or higher doses. Only MPs in stools were looked at in the present study. Neither MPs nor inflammatory markers in plasma, urine, or microbiota alterations were measured. Due to the carefully controlled indoor/outdoor environment in which the experiment was conducted, the experiment did not reflect daily exposure, chronic exposure, or differences in dietary habits or environmental contaminants in the general population.

## 5. Conclusions

In addition to demonstrating the potential of PCC as a “capturing biopolymer” for MPs in the gastrointestinal tract, this preliminary study laid the groundwork for larger studies with a longer sampling window (>12 h) and multiple comparison correction. It could be shown that PCC selectively retains MPs preferentially in the context of surface chemistry and structure (fibers vs. fragments, stereomicroscope-SEM-µFTIR analysis). Ultimately, MPs with a high H-bond density or more rigid structures (such as CE and PF) were retained for longer periods of time, while polymers with relatively smooth surfaces and functional groups that could interact through hydrogen bonding and electrostatic interactions (such as PE, PET, and RA) were excreted more easily. This “selective polishing” of MPs may help future dietary regimes to lessen the internal MP burden in humans. For the first time, this initial study demonstrates that a single dose of PCC (0.8 g) given to humans dramatically raises MP excretion after standardized SM. PCC has been shown to have the ability to reduce systemic exposure to MPs, just in a meal. Future research is guaranteed, with a minimum sample size of thirty and a collection period beyond 48 h. It is important to use multiple comparison correction in addition to evaluating inflammatory and microbial indicators.

## 6. Patents

Patent: P024280IT-01/CAT/Im “COMPOUNDS FOR BINDING OF MICROPLASTICS”, 2024.

## Figures and Tables

**Table 1:** General characteristics of volunteers: mean ± SD.

Subject Code	Gender (M/F)	Age (Years)	Body Weight (kg)	Height (m)	BMI (kg/m^2^)
*P* *01*	M	56	98	1.85	28.6
*P02*	M	41	87	1.82	26.3
*P03*	M	28	108	1.80	33.3
*P04*	M	20	61	1.63	23.0
*P05*	M	25	76	1.77	24.3
*P06*	M	19	86	1.84	25.4
*P07*	M	19	65	1.79	20.3
*P08*	F	33	52	1.64	19.3
*P09*	F	53	65	1.69	22.8
*P10*	F	18	59	1.73	19.7
AVERAGE		31	76	1.76	24.3
SD		14	18	0.10	4.4

**Table 2:** MP content in foods taken in the evening before stool collection (mean of 3 replicates).

Food Type	Mineral Water	Boiled Pasta	Tomatosauce	Extra Virgin Olive Oil	Parmesan Cheese	BreadIntegral	Tuna	Potatochips	Banana	Total
g or mL	250	180	30	5	5	35	80	25	120	730
MPs amounts	82	113	21	3	3	25	35	15	62	359

**Table 3:** Percentage of MPs size (µm) of fibers and fragments in SM (mean of 3 replicates).

**MPs (µm)**	20–100	≥100–250	≥250–500	≥500
**Fibers (%)**	9.0	48.0	41.0	2.0
**Fragments (%)**	16.0	53.0	28.0	3.0

**Table 4:** MPs recovered in stool samples in Phase 1 and Phase 2 after treatment with PCC.

Subject Code	Stool Quantity (g)	MPs Phase 1 Recovered	Stool Quantity (g)	MPs Phase 2 Recovered
	Phase 1		Phase 2	
*P01*	95.8	632	96.5	926
*P02*	92.4	702	94.3	1094
*P03*	89.6	717	95.1	1065
*P04*	87.4	454	90.9	618
*P05*	91.8	716	89.2	1035
*P06*	88.9	658	94.4	1001
*P07*	89.5	734	96.6	1140
*P08*	74.6	507	84.1	807
*P09*	83.8	788	86.9	1112
*P10*	79.4	635	83.4	851
AVERAGE	87.0	654.0	91.0	965
SD	6.4	103.8	5.0	165.0

**Table 5:** Percentages of the different MPs in SM and stool in before (Phase 1) and after (Phase 2) PCC administration: mean ± SD or range in 5 g samples.

MPs	In SM (%) 3 Samples	Phase 1 (%) Mean of 10 Samples	Phase 2 (%) Mean of 10 Samples
Acrylic Fiber	1	1.0 ± 0.5	-
CE	10	12 ± 3.3	5.0 ± 2.8
ER	4	4.0 ± 0.8	2.0 ± 1.6
PAN	1	1.0 ± 0.7	2.0 ± 0.7
PA	6	5.0 ± 0.8	5.0 ± 1.1
PTFE	1	1.0 ± 0.5	-
PF	4	4.0 ± 2.9	-
PE	11	13 ± 5.1	17 ± 4.4
PEI	5	4.0 ± 0.8	3.0 ± 1.2
PP	9	8.0 ± 2.3	5.0 ± 1.8
PES	11	14 ± 3.4	15 ± 3.8
PET	7	7.0 ± 3.5	10 ± 2.7
PEUR	1	1.0 ± 0.5	-
RA	28	25 ± 4.1	33 ± 4.7
PAM *	0	-	2.0 ± 1.6
PS *	0	-	2.0 ± 1.5
**TOTAL**	100	100	100

* T hese types of MPs were not present in SM.

**Table 6:** MPs of different origin in SM, Phase 1 and Phase 2, MPs: mean values ± SD in 5 g samples.

MPs Type	SM (3 Samples)	Phase 1 (10 Cases)	Phase 2 (10 Cases)	*p* Value ^a^ Phase 1 vs. Phase 2
	Mean	Mean ± SD	Mean ± SD	
Acrylic fiber	4	4.0 ± 3.7	1.0 ± 2.9	NS
CE	36	81 ± 28.1	50 ± 27.0	<0.05
ER	14	23 ± 5.6	17 ± 14.3	NS
PAN	7	5.0 ± 4.4	16 ± 6.9	<0.05
PA	22	33 ± 7.7	45 ± 11.6	<0.05
PTFE	4	3.0 ± 3.5	2.0 ± 4.5	NS
PF	14	26 ± 18.6	2.0 ± 3.3	<0.05
PE	39	88 ± 36.7	165 ± 50.7	<0.05
PEI	18	24 ± 5.7	24 ± 12.2	NS
PP	32	49 ± 14.3	54 ± 22.4	<0.05
PES	39	94 ± 28.3	145 ± 47.3	<0.05
PET	25	48 ± 24.3	98 ± 33.2	<0.05
PEUR	4	4.0 ± 3.4	3.0 ± 5.0	NS
RA	101	167 ± 39.8	326 ± 78.4	<0.05
PAM	0	2.0 ± 5.1	24 ± 19.1	<0.05
PS	0	2.0 ± 3.7	16 ± 11.9	<0.05
**T** **otal MPs**	**359**	**654 ± 103.8**	**965 ± 165.0**	**-**

^a^ = U Mann–Whitney; NS: Not statistically significant.

**Table 7:** Total MPs as fibers, fragments, and films in 5 g of stool for each volunteer during Phase 1 and Phase 2.

Subject Code	Phase 1			Phase 2		
	Fibers	Fragments	Films	Fibers	Fragments	Films
*P1*	21	12	0	33	15	0
*P2*	30	8	0	47	9	1
*P3*	31	10	0	36	19	1
*P4*	18	7	1	21	10	4
*P5*	24	12	2	36	18	5
*P6*	25	13	0	30	18	4
*P7*	27	14	0	37	18	4
*P8*	21	10	2	33	14	1
*P9*	31	16	0	42	21	1
*P10*	26	13	2	34	16	1
Mean	25	12	1	35 ^a^	16 ^a^	2 ^a^
SD	4.5	2.8	0.9	6.9	3.9	1.8
**Total (%)**	**68**	**31**	**1**	**66**	**30**	**4**

^a^ = U Mann–Whitney Phase 1 vs. Phase 2, *p* < 0.05.

**Table 8:** MP sizes as percentages and total amounts and stool in Phase 1 and Phase 2: mean for MP percentages and mean ± SD for the total MP in 5 g of stool.

Phase	Phase 1 [10] ^b^		Phase 2 [10] ^b^	
MP Size (µm)	% MPs	MP N ^c^	% MPs	MP N ^c^
>500	22	8.0 ± 2.2	20	11 ± 3.4 ^a^
250–500	42	16 ± 2.6	40	21 ± 4.5 ^a^
100–250	28	11 ± 4.0	32	17 ± 4.8 ^a^
20–100	9	3.0 ± 0.9	8	5.0 ± 1.7 ^ a^

^a^ = U Mann—Whitney Phase 1 vs. Phase 2, *p* < 0.05; ^b^ = number of samples considering that each case is in triplicate. ^c^ = Number of particles with no distinction among fibers, fragments, and films.

## Data Availability

Data are contained within the article.

## Supplementary Materials

The following supporting information can be downloaded at: https://www.mdpi.com/article/10.3390/foods14132190/s1, Figure S1: Average percentage of the shape of MPs analyzed and quantified in SM; Figure S2: MPs recovered in stool samples of 10 volunteers, before (Phase 1, baseline) and after treatment (Phase 2, PCC); Figure S3: Percentage of MPs shape before and after PCC use; Figure S4: Percentage of MPs size before and after PCC use; Figure S5: Technical data sheet of PCC used in the present study.

## Abbreviations

The following abbreviations are used in this manuscript:

AFM-Raman	atomic force microscopy–Raman
BMI	body mass index
CE	cellulose
CNS	central nervous system
DDA	deacetylation degree
ENS	enteric nervous system
ER	epoxy resin
FS	food supplement
IAPS	International Agency for Pharma Standard Supplements
MPs	microplastics
MW	molecular weight
NPs	nanoplastics
PA	polyamide
PAM	polyacrylamide
PAN	polyacrylonitrile
PCC	*Procambarus clarkii*
PE	polyethylene
PEI	polyethylenimine
PES	polyester
PET	polyethylene terephthalate
PEUR	polyether urethane
PF	phenol formaldehyde resin
PP	polypropylene
PS	polystyrene
PTFE	polytetrafluoethylene/Teflon
RA	rayon
SEM	scanning electron microscope
SM	standardized meal
