# Supplementary material for: Preliminary Study on PCC-Chitosan’s Ability to Enhance Microplastic Excretion in Human Stools from Healthy Volunteers
Source: Foods. 2025 Jun 23;14(13):2190. doi: 10.3390/foods14132190 (PMC12248620; doi:10.3390/foods14132190)
Supplement: Supplementary file 1 [file foods-14-02190-s001.zip › Supporting Information .pdf]

# Supporting Information

Preliminary Study on PCC-Chitosan's ability to Enhance Microplastic Excretion in Human Stools from Healthy Volunteers

Claudio Casella [1], Umberto Cornelli [2], Santiago Ballaz[3], Martino Recchia[4], Giuseppe Zanoni [1] and Luis Ramos-Guerrero[4*]

1 Department of Chemistry, University of Pavia, Viale Taramelli 12, 27100, Pavia, Lombardy, Italy; icarocus@gmail.com, gz@unipv.it
2 School of Medicine, Loyola University, Chicago, IL 60660, (USA) and Milan, Lombardy, Italy; ucornelli@gmail.com
3 Faculty of Health Sciences, Universidad del Espiritu Santo, Samborondón P.O. Box 09-01-952, Guayas, Ecuador; sballazg@gmail.com
3 Mario Negri Alumni, Milan, Lombardy, Italy; statmed@hotmail.com
4 Grupo de Investigación en Bio-Quimioinformática, Carrera de Ingeniería Agroindustrial, Facultad de Ingeniería y Ciencias Aplicadas, Universidad de Las Américas (UDLA), Quito 170513, Ecuador; luis.ramos.guerrero@udla.edu.ec

* Correspondence: luis.ramos.guerrero@udla.edu.ec

**Figure S1.** Average percentage of the shape of MPs analysed and quantified in SM

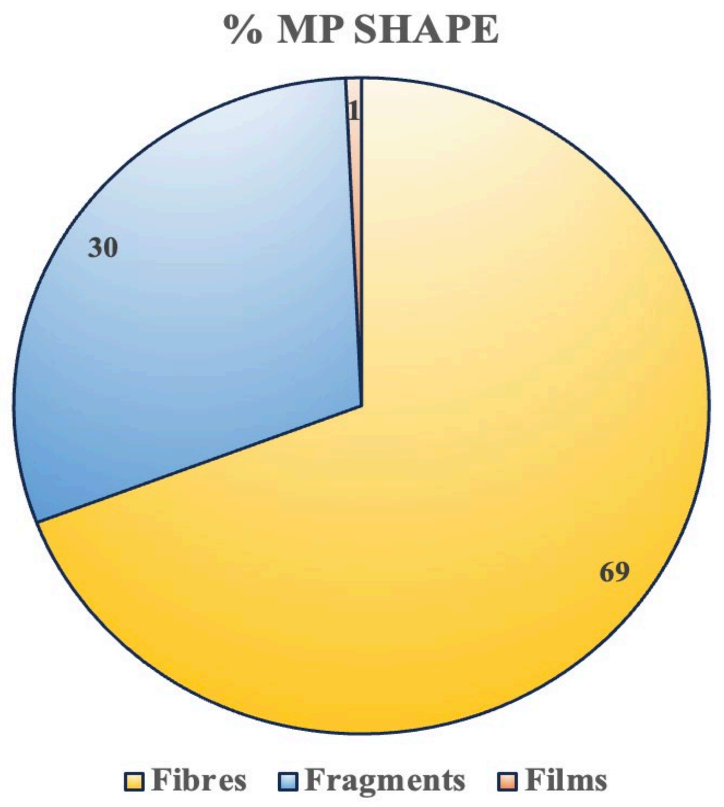


**Figure S2.** MPs recovered in stool samples of 10 volunteers, before (Phase 1, baseline) and after treatment (Phase 2, PCC)

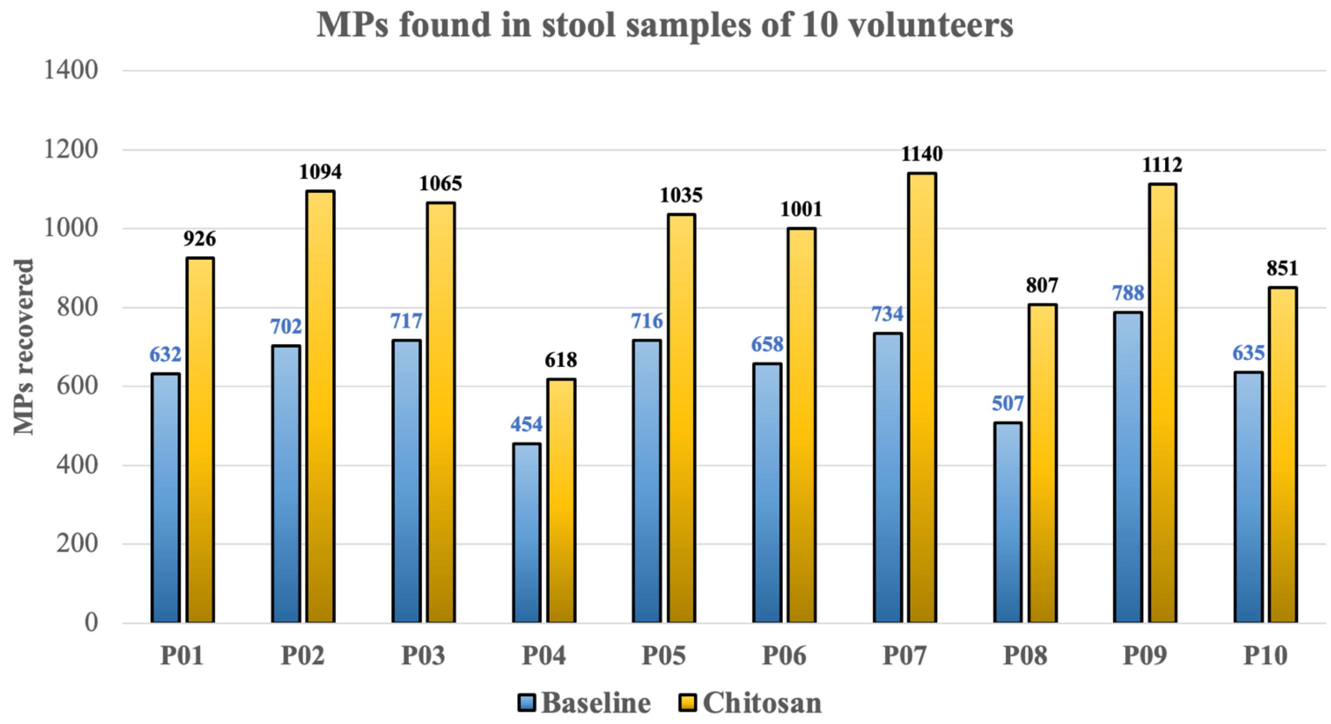

**Figure S3:** Percentage of MPs shape before (Baseline Phase 1) and after PCC use (Phase 2)

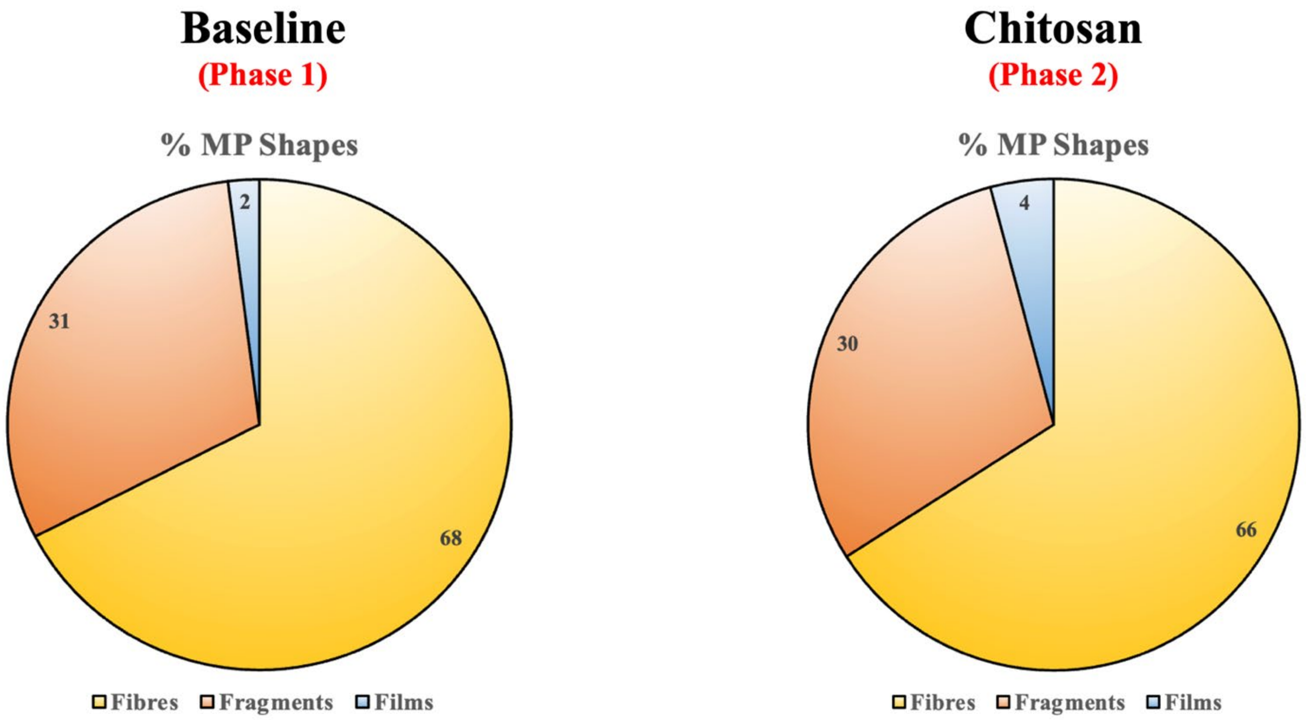


**Figure S4:** Percentage of MPs size before (Baseline Phase 1) and after PCC use (Phase 2)

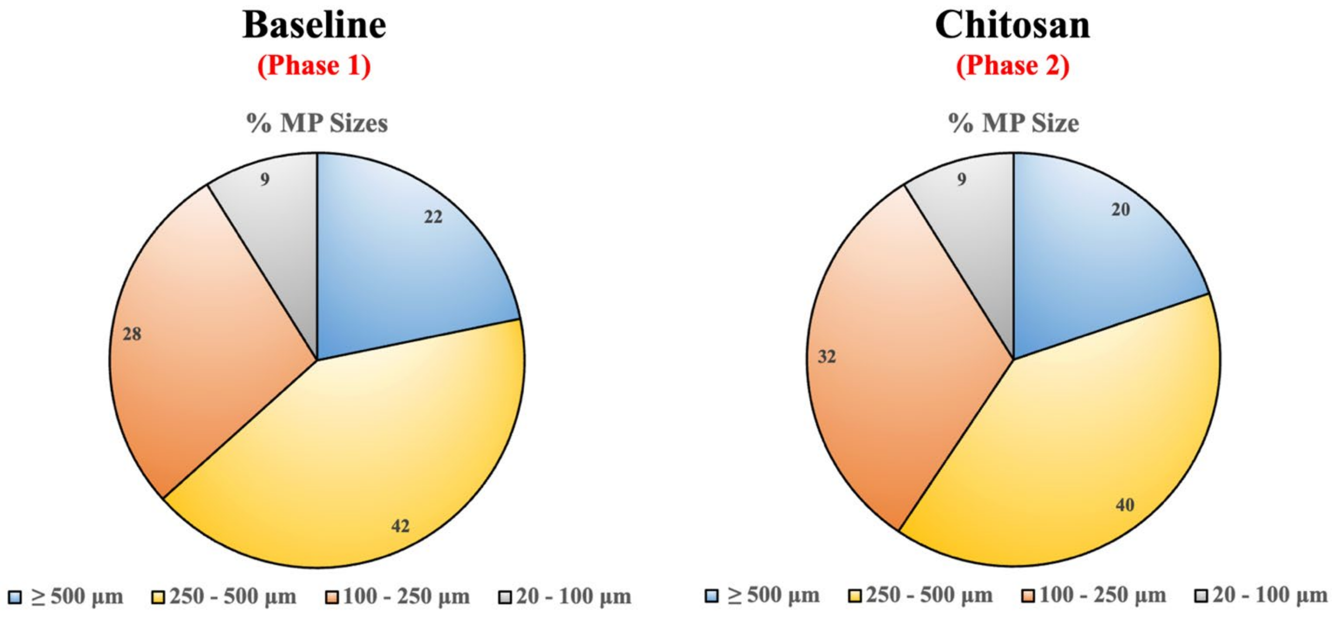

**Figure S5:** Technical data sheet of PCC used in the present study

| NUTRATRADE S.r.l. | | TECHNICAL DATA SHEET | | | |
|---|---|---|---|---|---|
| Name **Chitosan** | | SOURCE: **PROCAMBARUS CLARKII** | | | |
| Issued by | *QA* | Approved by | *Riccardo Cultraro* | Date **15/09/2023** | Rev. **5** |

| **Product Name** | Chitosan | **Solvent Used** | NA |
|---|---|---|---|
| **Country Origin** | China | **Grade** | Food ☒ Feed ☐ Pharma ☐ |
| **CAS No.** | 9012-76-4 | **Molecular Formula** | $C_{56}H_{103}N_9O_{39}$ |
| **Part No.** | 4.010-126.0 | **Shelf life** | 24 months when properly stored |
| **Packing** | Paper-drums and two plastic-bags inside, food contact material compliant with EC Reg. 1935/2004 and EU Reg. 10/2011 | **Storage** | Store in cool & dry place. Do not freeze. Keep away from strong light and heat. |

| **Analysis Item** | **Specification** | **Method** | **Provided on delivery** |
|---|---|---|---|
| Deacetylated Degree | ＞90% | NA | CoA |
| DER | NA | NA | NA |

| **Chemical and Physical Characteristics** | | | |
|---|---|---|---|
| **Analysis Item** | **Standard value** | **Method** | **Provided on delivery** |
| Appearance | Powder | Visual | DoC |
| Color | White to light yellow | Visual | DoC |
| Odor& Taste | Characteristic | Organoleptic | DoC |
| Identification | Positive | NA | DoC |
| Excipient | NA | NA | NA |
| Loss on drying | ≤10.0% | Ph.Eur.9.0<2.2.32> | CoA |
| Total ash | ＜1% | Ph.Eur.9.0<2.4.16> | CoA |
| Solubility in Water | NA | Organoleptic | DoC |
| Solubility in oil | NA | NA | DoC |
| Apparent density | NA | Eur.Ph.<2.9.34> | CoA |
| Tapped density | NA | NA | CoA |
| Solubility((in 1% Acetic Acid )) | ＞99.0% | NA | CoA |
| Viscosity | 90-130 mPa·s(cP) | NA | CoA |
| Sieve analysis | 95% through 100 mesh | USP39<786> | DoC |
| Arsenic (As) | NMT 1 ppm- Reg.EU 2023/915 | Ph.Eur.9.0<2.2.58>ICP-MS | DoC |
| Cadmium (Cd) | NMT 1 ppm- Reg.EU 2023/915 | Ph.Eur.9.0<2.2.58>ICP-MS | DoC |
| Lead (Pb) | NMT 3 ppm- Reg.EU 2023/915 | Ph.Eur.9.0<2.2.58>ICP-MS | DoC |
| Mercury (Hg) | NMT 0.1 ppm - Reg.EU 2023/915 | Ph.Eur.9.0<2.2.58>ICP-MS | DoC |
| Heavy Metals | NMT 10 ppm- Reg.EU 2023/915 | Ph.Eur.9.0 <2.4.8> | DoC |
| Pesticides Residues | Conform Reg.(EC) .396/2005 and amendments. | Gas Chromatography | DoC |
| Solvent Residues | Conform Eur.Ph. 9.0 <5,4 > and EC Dir. 2009/32 | Ph.Eur.9.0<2.4.24> | DoC |
| Hydrocarbons PAHs | ≤ 50 ppb -Reg.EU 2023/915 | GC-MS | DoC |
| Benzo(a)pyrene | ≤ 10 ppb -Reg.EU 2023/915 | GC-MS | DoC |
| Radioactivity | ≤ 600 Bq/Kg -Reg. EC 1048/2009 | NA | DoC |
| Aflatoxin B1 | ≤ 5 ppb - Reg.EU 2023/915 | Ph.Eur.9.0<2.8.18> | DoC |
| Aflatoxins Σ B1, B2, G1, G2 | ≤ 10 ppb - Reg.EU 2023/915 | Ph.Eur.9.0<2.8.18> | DoC |

Nutratrade

YOUR TRUST IS OUR SUCCESS®

| **Microbiological Characteristics** | | | |
|---|---|---|---|
| Total aerobic Count | ≤1000 cfu/g | Ph.Eur.9.0<2.6.12> | DoC |
| Yeast/Moulds | ≤100 cfu/g | Ph.Eur.9.0<2.6.12> | DoC |
| *Enterobacteriaceae* | ≤100 cfu/g | Ph.Eur.9.0<2.6.31> | DoC |
| *Escherichia coli* | Absent in 1 g | Ph.Eur.9.0<2.6.31> | DoC |
| *Salmonella* spp. | Absent in 25 g | Ph.Eur.9.0<2.6.31> | DoC |
| *Listeria monocytogenes* | Absent in 25 g | Ph.Eur.9.0<2.6.31> | DoC |
| *Staphylococcus aureus* | Absent in 1 g | Ph.Eur.9.0<2.6.31> | DoC |

| **Other product characteristics** | | |
|---|---|---|
| Irradiation | No Irradiation | DoC |
| GMO | Product No-GMO (Reg.1829/2003-1830/2003 EC) | DoC |
| Allergens | Crustaceans products thereof: Chitosan | DoC |
| Food Additives | Free (Reg. EU 1333/2008 and amendments) | DoC |
| BSE/TSE | Free | DoC |
| Melamine | Free, No melamine (Reg.EU 2023/915) | DoC |
| Pyrrolizidine alkaloids | In compliance with Reg.EU 2023/915 | DoC |
| Nanomaterials | Absent in compliance with Reg CE 1169/2011 | DoC |
| Ethylene oxide | No Ethylene oxide | DoC |
| Suitable for Vegan | No | DoC |

*DoC-Declaration of Conformity
*CoA-Result on CoA
